# Semiautomatic segmentation of the kidney in magnetic resonance images using unimodal thresholding

**DOI:** 10.1186/s13104-016-2292-z

**Published:** 2016-11-17

**Authors:** Martin Sandmair, Matthias Hammon, Hannes Seuss, Ragnar Theis, Michael Uder, Rolf Janka

**Affiliations:** Department of Radiology, Universitätsklinikum Erlangen, Friedrich-Alexander-Universität Erlangen-Nürnberg (FAU), Maximiliansplatz 1, 91054 Erlangen, Germany

**Keywords:** Kidney volume, Magnetic resonance, Unimodal thresholding, Intensity histogram, Probability distribution

## Abstract

**Background:**

Total kidney volume (TKV) is an important marker for the presence or progression of chronic kidney disease, however, routine ultrasonography underestimates renal volume to a high and varying degree.

**Objective:**

The aim of this work was to adapt and evaluate a semi-automatic unimodal thresholding method for volumetric analysis of the kidney in native T2-weighted magnetic resonance (MR) images.

**Methods:**

In a group of healthy volunteers (n = 24; 48 kidneys), we defined a region of interest (ROI) by manually tracing the outline of the kidney in every MR image. An automatic unimodal thresholding algorithm with visual feedback was applied to the probability distribution function of voxel intensities in the ROI to remove intrarenal non-parenchyma volume. For comparison, reference volumes were created by manual segmentation. Intra- and inter-observer reliability was evaluated.

**Results:**

There was a small, significant mean difference of 1.5 ml between semi-automatically and manually segmented TKV (p = 0.009, 95% CI [0.4, 2.7]). While intra-observer reliability was good (mean difference 2.9 ml, p < 0.01, 95% CI [1.5, 4.2]) there was a small but significant mean difference of 4.8 ml (p < 0.01, 95% CI [3.6, 5.9]) between the TKV results of different observers. Reference volume correlations were excellent (r = 0.97–0.98). Semi-automated segmentation was significantly faster than manual segmentation; mean difference = 234 s [91–483 s]; p < 0.05. Automatic unimodal thresholding removed a considerable mean volume of 18.7 ml (13.1%) from the coarse manual pre-segmentations.

**Conclusions:**

Unimodal thresholding of native MR images is a robust and sufficiently reliable method for kidney segmentation and volumetric analysis. The manual pre-segmentation can be done by non-experts with little introduction.

**Electronic supplementary material:**

The online version of this article (doi:10.1186/s13104-016-2292-z) contains supplementary material, which is available to authorized users.

## Background

Chronic kidney disease (CKD) is a public health problem with a growing incidence in aging populations [1, 2]. A change in kidney volume is a potential marker for the presence or progression of CKD. Kidney volume also helps to determine therapy and invasive diagnostics, e.g. kidney biopsy [3]. In ultrasonography, TKV is usually calculated by a variation of the ellipsoid formula. Unfortunately, this approximation significantly underestimates the kidney volume and suffers from poor repeatability [3, 4]. Therefore more accurate, reproducible and fast alternatives for determining TKV are desirable. Magnetic resonance imaging (MRI) uses a tomographic method, is non-invasive and does not expose patients to ionizing radiation, contrary to computed tomography. It should be acknowledged that MRI-based examinations are limited by the relatively high costs, the partly limited access to scanners (e.g., compared to ultrasound), some contraindications (e.g., subjects with some types of metal implants or pacemakers), and the potentially reduced convenience and tolerability for patients (e.g., claustrophobia). Due to the fact that the acquisition of MR images takes longer than the acquisition of CT images motion artifacts may occur, especially when imaging breath-dependent organs such as the kidney.

Manual slice by slice segmentation of the kidney parenchyma, excluding e.g. intrarenal vessels, calyces, cysts, is time intensive and user-dependent. There are various approaches that fully or partly automate the process [5]. Fully automatic segmentation of the kidney in MR images is a challenging problem, owing to noise, low contrast, artifacts and the highly variable shape of the organ. Examples for state of the art approaches include methods based on the generation of probability maps [6] and neural networks [7]. Semiautomatic solutions often combine local adaptive algorithms, which detect edges and contours in an image, with an interactive mouse tool. The user interactively ‘brushes’ the kidney with the tool and achieves faster segmentation. Local adaptive algorithms have parametric properties that need to be adjusted manually by trial and visual feedback, depending on image quality, MR sequence and, most importantly, the shape and structure of the kidney itself. This leads to operator dependence in adjusting these properties and can be time intensive.

The aim of this work was to develop and evaluate a semi-automatic unimodal thresholding method for volumetric analysis of the kidney in native T2-weighted MR images. Unimodal thresholding has first been described by Rosin et al. [10] and works with any image that has a unimodal intensity distribution. Rosin et al. [11] evaluated images of various modalities with good results, including CT angiograms. There also exists an adaptation for volumetric segmentation of 3D breast images acquired with cone-beam computed tomography. Our adaptation is based on the distribution of the voxel intensities in a rough, manually pre-segmented region of interest (ROI) of the kidney. To our knowledge, the proposed implementation is the first in the MRI domain.

## Methods

### Volunteers

In this study, 24 healthy subjects were chosen (11 males, 13 females). The mean age was 26 years (range: 21–41 years). The body mass index of all subjects was on average 21.8 kg/m^2^ (range: 18.9–24.8 kg/m^2^). The study recruitment process began in July 2015 and was conducted using an advertisement sent to our circle of acquaintances. The informed consent documents delineated the MR exam and informed the volunteer that data collected during the exam could be used for research purposes. Informed consent forms were signed by every volunteer in the study. The study was executed according to the Declaration of Helsinki and ‘good clinical practice’ (GCP) guidelines. The institutional review board of the University Hospital Erlangen/Germany approved the study.

### MR examinations

In 19/24 MR examinations, we used a 1.5 Tesla MR Scanner (Magnetom Avanto, Siemens, Erlangen, Germany). The remaining 5/24 examinations were acquired on a 3 Tesla MR scanner (Magnetom Verio, Siemens, Erlangen, Germany). The volunteers were examined in supine position with a standard abdominal phased array coil (Siemens, Erlangen, Germany). For each subject, a volume containing both kidneys was scanned using a T2 turbo spin echo (TSE) sequence with prospective acquisition correction (PACE) in axial orientation and the following parameters: TR 4400 ms, TE 88 ms, bandwidth 260 Hz/px, acquisition matrix 328 × 288 px, slice thickness 4.4 mm, in-plane resolution 1.0 × 1.0 mm.

### Manual pre-segmentation

For the manual pre-segmentation we used the Multi-image Analysis GUI (Mango) image processing system.^1^ Mango was selected because it is easy to use and employs the standard Neuroimaging Informatics Technology Initiative (NIfTI) file format for saving image volumes and ROI.

### Unimodal thresholding

The unimodal thresholding algorithm was implemented by one of the authors (M.S.) as a custom script in Python, version 2.7^2^ using the Scipy ecosystem, Numpy,^3^ nibabel^4^ and scikit-image^5^ libraries. Spyder, a scientific development environment,^6^ and part of the Anaconda software suite^7^ was selected for ease of use.

**Fig. 1:**
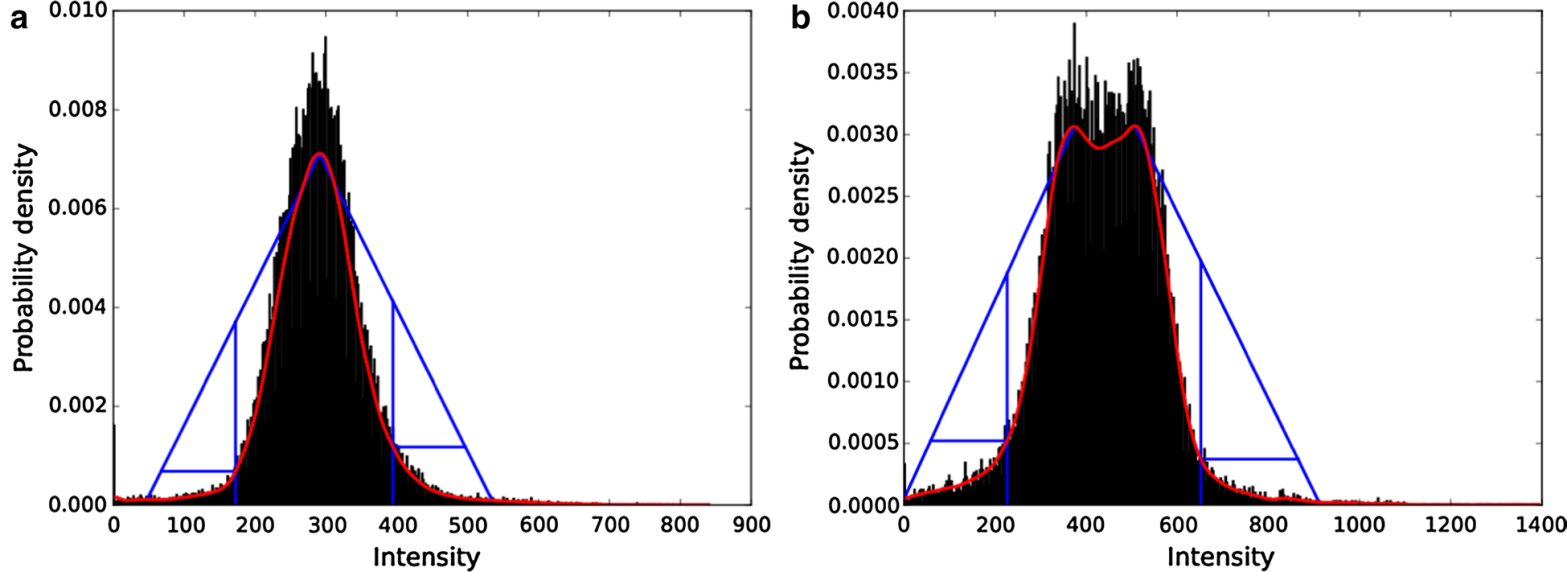
**a** Intensity histogram for one kidney, fitted PDF and delineations of the unimodal thresholding algorithm, **b** bimodal intensity histogram fitted PDF and delineations of the unimodal thresholding algorithm with two maxima

**Fig. 2:**
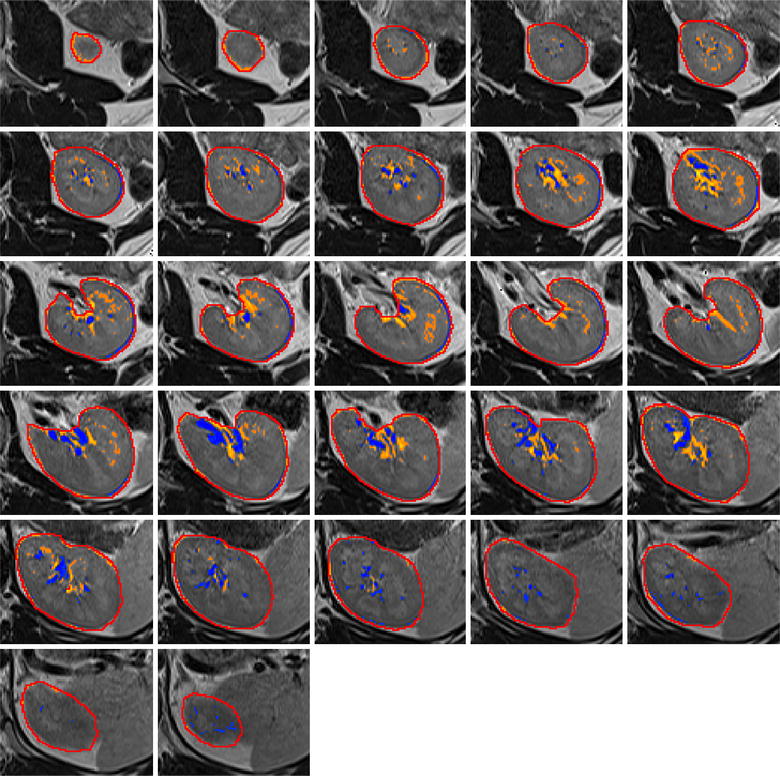
Kidney region of interest (axial slices). *Red border* represents the manual pre-segmentation. *Blue hues* depict voxels below the lower threshold. *Orange hues* depict voxels above the upper threshold

The script takes an abdominal MRI and a ROI around one kidney, both in NifTI-format, as input. From all voxel intensities within the ROI, a probability distribution function (PDF) is generated with a kernel-density estimate using a Gaussian kernel^8^ and Scott’s method [8]. The maxima of the PDF are detected automatically by a peak-seeking algorithm.^9^ If there is more than one maximum, the user has the choice to depart from the default, which is the global maximum of the PDF. Two points on the PDF with a distance of one standard deviation left and right of its maximum are calculated. Straight lines are drawn from the peak to these points. The area of a right triangle, its hypotenuse part of the respective line and its right vertex on the PDF, is maximized—this is equivalent to the maximum perpendicular distance between the PDF and the line. The upper and lower threshold is determined by the right vertices on the PDF (Fig. 1a). For visual feedback in all slices of the kidney volume the manual ROI and all voxels within the upper and lower threshold are shown as a colored line and colored area (Fig. 2).

The volume of all voxels within the threshold is automatically calculated and exported into a SQLite database created with SQLite Manager.^10^^,^ 11

### Reference volume

A manual segmentation was performed with Photoshop (Version CS6, Adobe Systems, San Jose, CA, USA). The entire kidney parenchyma was segmented from the surrounding tissues manually on the T2-weighted MR images using knowledge about the shape, location and structure of the kidney. The contours of both kidneys were carefully drawn manually in each slice for each volunteer. The manual segmentation was performed by a medical student. A board-certified radiologist [6 years of work experience (M.H.)] verified and corrected the segmentation where necessary. These delineations were considered as the reference volume.

### Statistical analysis

The statistics in the study were calculated using R, version 3.2.1.^12^ For the inter-observer variation study, manual segmentation of all 48 kidneys was performed by two independent observers, one medical postgraduate and one layperson. An intra-observer variation study was performed by comparing two segmentation groups of all 48 kidneys by the same observer (medical postgraduate) with a 6 months minimum time difference. These results were compared to a reference volume, of all 48 kidneys, obtained by a purely manual segmentation (see above) by another independent observer. All observers were blind to the others results. Correlations were calculated using Pearson’s product-moment correlation coefficient. Statistical differences between groups were compared with a paired t test where p < 0.05 was considered significant. Comparisons were performed with a linear regression analysis and the Bland–Altman method [9].

## Results

### Voxel histogram and probability distribution function (PDF)

**Fig. 3:**
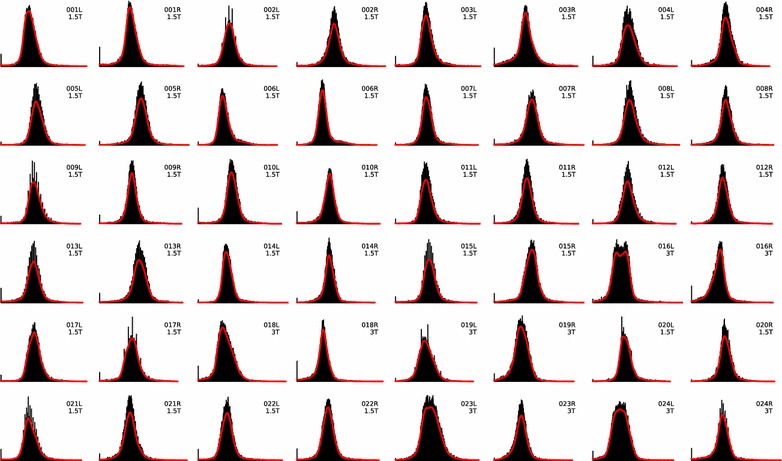
Voxel histogram with fitted PDF for all 48 kidneys

Except for two kidneys, the PDFs are unimodal which means that they have a single global maximum (Fig. 3). Kidneys 016L and 023L have two local maxima (bimodal). The default method uses only the global maximum; local maxima are ignored. Choosing the maxima near the respective lines as their peak points (Fig. 1b) and comparing the result to the default we found a maximum volume difference of 1.2 ml for kidney 016L (observer 1) and of 0.4 ml for kidney 023L (observer 2). We consider these differences negligible. The results reported below are based on the automatic global maximum method.

### Visual evaluation

Throughout the evaluation, the areas marked by the automatic threshold corresponded well to anatomical features like vessels and calyxes (Fig. 2).

### Numerical results

Using unimodal thresholding, the mean TKV was 143.2 ± 29.0 ml; 146.3 ± 28.0 ml for the left kidney and 140.1 ± 29.8 ml for the right kidney; 157.1 ± 26.2 ml for male and 131.4 ± 25.9 ml for female subjects; see Table 1.

**Table 1:** Comparison of mean renal volume (ml) for three unimodal segmentation groups and one manual reference group

			Mean total kidney volume (ml) ± SD				
			Total	Left kidney	Right kidney	Male	Female
Semiautomatic segmentation (unimodal thresholding)	Observer 1	1	146.2 ± 29.8	150.2 ± 29.1	142.2 ± 30.5	160.9 ± 26.4	133.7 ± 27.0
		2	143.3 ± 29.1	147.3 ± 28.0	139.4 ± 30.2	157.2 ± 26.4	131.6 ± 26.3
	Observer 2		140.0 ± 28.3	141.4 ± 27.3	138.7 ± 29.9	153.2 ± 26.5	128.9 ± 25.2
	Total		143.2 ± 29.0	146.3 ± 28.0	140.1 ± 29.8	157.1 ± 26.2	131.4 ± 25.9
Manual segmentation (reference volume)			141.7 ± 28.5	143.7 ± 27.5	139.6 ± 29.9	156.5 ± 24.4	129.1 ± 25.9

There was a small, significant mean difference of 1.5 ml between the volumes acquired by semi-automated segmentation and the reference volume (95% CI [0.4, 2.7], N = 144, p < 0.01, paired t test). The mean absolute difference (MAD) was 5.5 ml.

Repeated measurements by the same observer (intra-reader reliability) showed a small, significant variability; the mean difference was 2.9 ml (95% CI [1.5, 4.2], N = 48, p < 0.01, paired t test), the MAD was 4.3 ml.

To evaluate inter-reader reliability, both segmentation groups of observer 1 were compared to one segmentation group of observer 2. We found a small, significant mean difference of 4.8 ml (95% CI [3.6, 5.9], N = 96, p < 0.01, paired t test) and a MAD of 6.0 ml. A detailed summary is shown in Tables 2 and 3.

**Table 2:** Accuracy and reliability of the semiautomatic segmentation

		N	Min	5th percentile	Mean	95th percentile	t	Max	SD	p (2-tailed)	MAD
Accuracy (difference to the reference volume)	Observer 1 (1)	48	−11.4	2.4	4.5	6.7	4.3	30.4	7.4	<0.01	6.6
	Observer 1 (2)	48	−7.7	0.1	1.7	3.2	2.2	15.3	5.4	<0.05	5.4
	Observer 2	48	−15.5	−3.6	−1.6	0.3	−1.7	11.2	6.7	0.095	4.4
	Total	144	−15.5	0.4	1.5	2.7	2.6	30.4	7.0	<0.01	5.5
Inter-observer reliability	Observer 1 (1)/observer 2	48	−13.9	4.3	6.2	8.1	6.5	24.8	6.5	<0.01	7.3
	Observer 1 (2)/observer 2	48	−7.6	1.9	3.3	4.7	4.9	13.9	4.7	<0.01	4.4
	Total	96	−13.9	3.6	4.8	5.9	7.9	24.8	5.9	<0.01	6.0
Intra-observer reliability	Observer 1 (1)/observer 1 (2)	48	−6.3	1.5	2.9	4.2	4.4	15.1	4.5	<0.01	4.3

**Table 3:** Correlation and absolute mean difference of three unimodal segmentation groups to the reference volume (ml)

		Correlation to the reference volume				Mean difference to the reference volume (ml) ± SD				
			95% CI	t	p (2-tailed)	Total	Left	Right	Male	Female
Observer 1	1	0.97	[0.94, 0.98]	26.5	<2.2e−16	4.5 ± 7.4	6.5 ± 7.9	2.6 ± 6.4	4.4 ± 9.5	4.7 ± 5.1
	2	0.98	[0.97, 0.99]	36.0	<2.2e−16	1.7 ± 5.4	3.5 ± 5.7	−0.2 ± 4.4	0.7 ± 6.1	2.5 ± 4.6
Observer 2		0.97	[0.95, 0.99]	28.0	<2.2e−16	−1.6 ± 6.7	−2.3 ± 6.9	−0.9 ± 6.4	−3.4 ± 7.2	−0.2 ± 5.9

**Fig. 4:**
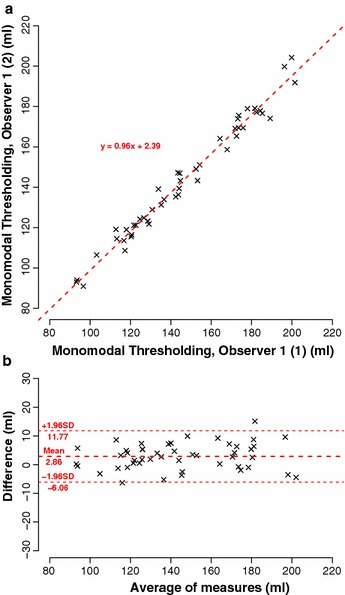
Intra-observer reliability: comparison between observer 1 (1) and observer 1 (2), 48 kidneys. **a** Correlation. **b** Corresponding Bland–Altman plot

Comparing the results acquired by the same observer, we found excellent correlation (r = 0.99, t = 44.1, p < 0.01, 95% CI [0.98, 0.99]) and excellent agreement (mean value of differences = 2.9 ± 4.5 ml, 95% CI [−6.1, 11.8]) (see Fig. 4).

**Fig. 5:**
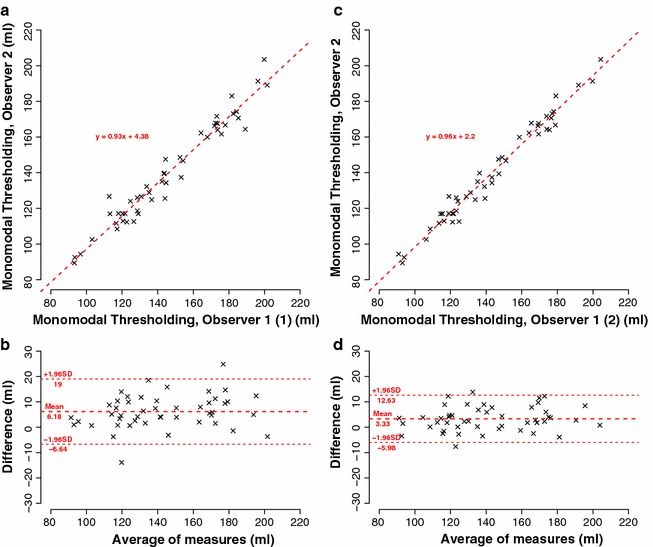
Inter-observer reliability: **a** Correlation of observer 1 (1) to observer 2, 48 kidneys. **b** Corresponding Bland–Altman plot. **c** Correlation of observer 1 (2) to observer 2, 48 kidneys. **d** Corresponding Bland–Altman plot

Comparing the results acquired by different observers, we also found excellent correlation (observer 1 (1)/observer 2: r = 0.98, 95% CI [0.96, 0.99], p < 0.001; observer 1 (2)/observer 2: r = 0.99, 95% CI [0.98, 0.99], p < 0.01) and good agreement (observer 1 (1)/observer 2: mean value of differences = 6.2 ± 6.5 ml, 95% CI [−6.6, 19.0], observer 1 (2)/observer 2: mean value of differences = 3.3 ± 4.7 ml, 95% CI [-6.0, 12.6]), see Fig. 5.

**Fig. 6:**
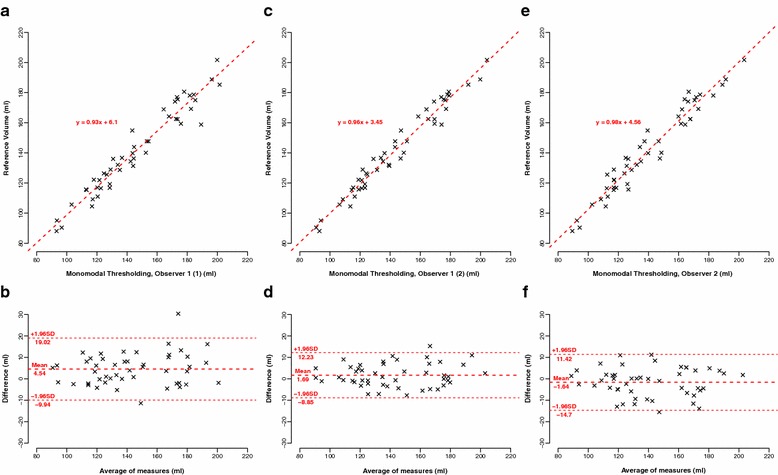
Comparison to the reference volume, 48 kidneys. **a** Correlation to observer 1 (1). **b** Corresponding Bland–Altman plot. **c** Correlation to observer 1 (2). **d** Corresponding Bland–Altman plot. **e** Correlation to observer 2. **f** Corresponding Bland–Altman plot

Comparing the three unimodal thresholding TK volumes to the reference volume, there was again excellent correlation (Table 3) and excellent agreement (Fig. 6). For the manual segmentation, the time required per kidney was 408 ± 105 s. The semi-automated segmentation took 174 ± 38 s and, therefore, was significantly faster.

The mean automatically removed volume of all observers was 18.7 ml (13.1%), shown in Table 4.

**Table 4:** Mean automatically removed volume for three unimodal segmentation groups (ml)

		Mean automatically removed volume (ml) ± SD				
		Total	Left	Right	Male	Female
Observer 1	1	18.1 ± 4.9	15.8 ± 4.2	20.5 ± 4.5	19.2 ± 4.8	17.3 ± 4.9
	2	19.3 ± 5.4	18.5 ± 5.9	20.0 ± 4.9	20.6 ± 5.3	18.1 ± 5.3
Observer 2		18.8 ± 5.9	16.2 ± 4.7	21.4 ± 5.9	20.0 ± 5.4	17.8 ± 6.2
Total		18.7 ± 5.4	16.8 ± 5.0	20.6 ± 5.1	19.9 ± 5.1	17.7 ± 5.5

## Discussion

The aim of this work was to develop and evaluate a fast, robust, accurate and reliable method for volumetric analysis of the kidney in native T2-weighted MR images. We developed an automatic algorithm to partition the PDF of the voxel intensities within a rough and manually acquired kidney ROI. This thresholding method of essentially unimodal distributions has been described and tested with images of various modalities [10]. It has also been successfully adapted for volumetric segmentation of 3D breast images acquired with cone-beam computed tomography [11].

It should be mentioned that the obtained thresholds are not fixed. They are case-dependent and vary with the peak location of the PDF derived from the ROI intensity histogram. This is an important advantage, because MR intensity is arbitrary and varies between subjects, scanners and imaging sessions. Of note, MR intensity normalization techniques are commonly based on the image histogram [12]. In our case the underlying assumptions are that the kidney parenchyma, consisting of a cortex and medulla, is the predominant tissue in the kidney ROI and has characteristic intensity values that are different from non-parenchyma tissue. This then shows as a peak in the intensity histogram of the ROI and the derived PDF. We used points on the PDF with a distance of one standard deviation left and right of its maximum rather than the left and right ends of the PDF. This empirical modification was necessary because, without it, right-sided outliers (high intensity) would lead to markedly higher upper thresholds and a systematically too high TKV. Also, the lower threshold would otherwise be influenced by the location of the maximum of the PDF.

The method was tested on 24 scans of healthy volunteers (48 kidneys). It removed a considerable mean volume of 13.1% from the manually pre-segmented ROI. On visual inspection this volume consisted predominantly of non-parenchyma tissue such as blood vessels and calyxes. Renal volume, listed in Table 1, is in good accordance with values reported in the literature [4]. Intra- and inter-observer reliability was good, and the results correlated well with a manually segmented reference volume.

The unimodal thresholding approach was proposed by Rosin et al. more than 15 years ago [10]. Since then, numerous approaches were described in the domain of MR image segmentation [5–7]. Our proposed method requires a rough manual pre-segmentation of the kidney. However, it affords significant time savings compared to a fine manual segmentation and could be used for generating training data for segmentation approaches that employ machine learning techniques. The necessary coarse manual pre-segmentation could be done by non-experts with little introduction. The end-results should be independent of the software used for this step. This could be advantageous for the evaluation of large data sets. Kidney volume was determined automatically at completion of the unimodal thresholding algorithm and we did not make any corrections. Our study has some limitations. The unimodal thresholding only removes voxels that differ somewhat from the predominant intensity within the manually pre-segmented ROI. If voxels with similar intensity (spleen, pelvis, vascular tissue, dull margins due to partial volume effects) are included in the pre-segmentation they will not be removed by the subsequent automatic unimodal thresholding. This leads to a false high TKV.

Additionally, if a lot of near isointense non-parenchyma voxels are included in the manual pre-segmentation the intensity distribution of the whole ROI could be altered in a way that leads to higher thresholds compared to a sufficiently accurate pre-segmentation, resulting in a systematic error towards a higher TKV. We did not see this error in our pre-segmentations (observer 2 is a layperson); therefore, we did not see this property having a considerable impact.

However, we saw small but significant systematic differences between observation groups (Table 2). We consider these to be mainly a manifestation of user dependability concerning the manual segmentation, which is the tendency of the user to include or exclude disputable volume that may or may not be part of the kidney. These differences were more pronounced for left kidneys than for right kidneys (Table 3), which is most likely due to the proximity of iso-intense spleen tissue. These systematic errors were sufficiently small for us to consider acceptable.

For the evaluation of our semiautomatic segmentation approach, we used images of healthy volunteers. Further research is necessary to show how the algorithm works with data of patients that could have a worse image quality, different intensity distributions within the kidney or lower contrast between renal parenchyma and surrounding tissue.

For the clinical setting a more interactive process would be desirable, such as unimodal thresholding executed every time the user draws a kidney contour in an image slice. For diseased kidneys it could be beneficial if the user had the ability to manipulate thresholds along the pdf with instant feedback.

Lastly, all of the software used is open source for research purposes. It stands to reason that this thresholding method is promising also for different tissues and different image modalities, as long as the intensity distribution of the voxels in the region of interest is nearly unimodal. The Python code for the implementation of the unimodal thresholding algorithm consists of a central script, several functions and sub functions. The Python code is available as additional material (Additional file 1).

## Conclusions

Unimodal thresholding of native MR images is a robust and sufficiently reliable method for kidney segmentation and volumetric analysis. The manual pre-segmentation can be done by non-experts and with a brief introduction. Future research includes the evaluation of kidney voxel distributions at different magnetic field strengths, the evaluation of alternative MR protocols, embedding of unimodal thresholding in a more interactive protocol and the investigation of patient data as well as large data sets.

## Additional file

**Additional file 1.** **unimodcode.zip:** a zipped folder containing the following files: **unimodscript.py**: the central script for the unimodal thresholding approach, **l03threshSTD.py**: the monomodal thresholding algorithm function, **l02shrink.py**: a function to remove most of the whitespace from a masked volume, **detect_peaks.py**: the peak detection function by Marcos Duarte, also available at https://github.com/demotu/BMC/blob/master/functions/detect_peaks.py, **example.nii.gz**: an anonymized abdominal MRI in NIfTI format, **example_left_kidney_preseg.nii.gz**: a manual pre-segmentation of the left kidney of the example MRI, done with the Multi-image Analysis GUI (MANGO), available at http://ric.uthscsa.edu/mango/, **example_right_kidney_preseg.nii.gz**: a manual pre-segmentation of the right kidney of the example MRI, done with the Multi-image Analysis GUI (MANGO), available at http://ric.uthscsa.edu/mango/, **LICENSE.txt**: the MIT license text, **readme.txt**: description of the included files, list of dependencies, instructions for installation and use.

## Abbreviations

CKD: chronic kidney disease
GCP: good clinical practice
GUI: graphical user interface
MAD: mean absolute difference
MR: magnetic resonance
MRI: magnetic resonance imaging
NIfTI: Neuroimaging Informatics Technology Initiative
PACE: prospective acquisition correction
PDF: probability distribution function
ROI: region of interest
TKV: total kidney volume
TE: time to echo
TR: time to repeat
TSE: turbo spin echo

## Footnotes

1 [http://ric.uthscsa.edu/mango] Mango. Accessed 10 June 2016.

2 [http://www.python.org] Python. Accessed 10 June 2016.

3 [http://docs.scipy.org/doc/] Numpy and Scipy Documentation. Accessed 10 June 2016.

4 [http://nipy.org/nibabel/] Nibabel. Accessed 10 June 2016.

5 [http://scikit-image.org/]Scikit-image. Accessed 10 June 2016.

6 [https://pythonhosted.org/spyder/] Spyder. Accessed 10 June 2016.

7 [http://docs.continuum.io/anaconda/index] Anaconda. Accessed 10 June 2016.

8 [http://docs.scipy.org/doc/scipy/reference/generated/scipy.stats.gaussian_kde.html] SciPy Kernel Density Estimate. Accessed 7 June 2016.

9 [https://github.com/demotu/BMC/blob/master/functions/detect_peaks.py] DetectPeaks. Accessed 10 June 2016.

10 [https://www.sqlite.org/] SQLite. Accessed 10 June 2016.

11 [https://addons.mozilla.org/en-us/firefox/addon/sqlite-manager/] SQLite Manager. Accessed 10 June 2016.

12 [https://www.r-project.org/] The R Project for Statistical Computing. Accessed 10 June 2016.
